# Case report: Fatal systemic embolism caused by early prosthetic valve endocarditis after Bentall surgery

**DOI:** 10.3389/fcvm.2022.1020186

**Published:** 2023-01-09

**Authors:** Shaofeng Wu, Xin Wang, Weidong Ren, Guang Song, Yang Hou, Haidi Hu, Xiaona Yu

**Affiliations:** ^1^Department of Ultrasound, Shengjing Hospital of China Medical University, Shenyang, China; ^2^Department of Radiation, Shengjing Hospital of China Medical University, Shenyang, China; ^3^Department of Vascular Surgery, Shengjing Hospital of China Medical University, Shenyang, China

**Keywords:** Bentall surgery, prosthetic valve endocarditis, systemic embolism, staphylococcus endocarditis, echocardiography

## Abstract

Prosthetic valve endocarditis (PVE) is a rare but dangerous complication of Bentall surgery and *Staphylococcus epidermidis* PVE involving multiple valves simultaneously during the early postoperative period has not been reported. A 42 year old patient admitted to intensive care unit with fever 1 month after aortic valve replacement (Bentall procedure). Echocardiography was of great diagnosis value and suggested large, mobile vegetations on both the prosthetic aortic valve and native tricuspid valve. The presence of *Staphylococcus epidermidis* was revealed by multiple blood cultures. Surgery was not performed because of the history of aortic valve replacement 1 month ago. He developed acute right femoral artery thromboembolism, multiple cerebral infarction and splenic infarction during hospitalization and died of cerebral infarction after being discharged. This case underlines that patients with early PVE may have poor prognosis and fatal systemic embolism should be aware of in PVE patients with large vegetations present with dyskinesia, abdominal pain, and limb numbness. The timely echocardiography and vascular ultrasound are primary and reliable diagnostic methods in this scenario.

## Highlights

– Early PVE is a rare but dangerous complication of aortic valve replacement.
– Soft tissue infection during the early postoperative period of Bentall surgery may lead to PVE, which can cause a poor prognosis.
– Echocardiography and vascular ultrasound are of great importance and value in the diagnosis of PVE.
– It is necessary to prevent postoperative infection because early PVE cannot be resolved by reoperation.

## Introduction

Bentall procedure is the gold standard treatment for patients with ascending aortic aneurysm and aortic valve stenosis or regurgitation (1). PVE is a rare but dangerous consequence after prosthetic valve implantation with an incidence of 0.3–1.2% per patient-year, and the risk for PVE is 1.7% within the first year after operation (2). *Staphylococcus epidermidis* infective endocarditis (IE) is rare within 1 year after valve replacement (3). We herein reported a sporadic case of Staphylococcus IE involving multiple valves and systemic embolisms 1 month after Bentall surgery in a 42-year-old man with Marfan’s syndrome.

## Case presentation

A 42-year-old man was admitted to the hospital due to unexplained chest pain and dyspnea. Echocardiography suggested Marfan’s syndrome, showing aortic root dilated up to 70 mm and moderate aortic regurgitation. Finally, the patient received aortic root replacement (Bentall surgery) to alleviate clinical symptoms. He has history of hypertension for 1 year. The patient recovered well from the surgery. However, he developed a perianal abscess in the following weeks.

**FIGURE 1:**
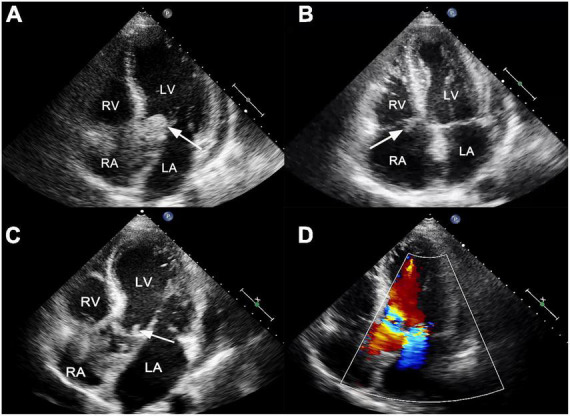
**(A)** Transthoracic echocardiogram showed a large, mobile vegetation on the ventricular side of the prosthetic valve (white arrow). **(B)** A vegetation was also visible on the tricuspid valve (white arrow). **(C)** Transthoracic echocardiogram reexamination showed residual vegetations on prosthetic aortic valves with reduced size (white arrow). **(D)** The new mitral valve moderate regurgitation. LA, left atrium; LV, left ventricle; RA, right atrium; RV, right ventricle.

He presented to the emergency department with persistent fever (38°C) and fatigue 1 month after surgery. The blood pressure was 109/71 mmHg, breathing rate was 16 breaths/min, pulse was 88 beats/min, and temperature was 36.5°C. Laboratory results showed white blood cell 19.6 × 10^9^/L, hemoglobin 95 g/L, platelet count 106 × 10^9^/L, C-reactive protein 195 mg/L, B-type natriuretic peptide 761.8 pg/ml, hypersensitivity troponin T 0.174 ng/ml. Electrocardiographic analysis revealed a first-degree atrioventricular block. A heart examination showed a murmur at the aortic area. Transthoracic echocardiogram (TTE) revealed a large (25 mm × 22 mm), iso-echogenic, irregular vegetation on the ventricular side of the prosthetic aortic valve (Figure 1A) winging back and forth during the cardiac cycle. In addition, a large (16 mm × 9 mm) tricuspid valve vegetation with obvious mobility was also presented (Figure 1B). IE was suspected according to the modified Duke criterion (4). Therefore, vancomycin (1,000 mg, every 12 h) and gentamicin (240 mg, every 8 h) were given for infection control. Rifampin was orally taken (0.3 g, every 8 h). In addition, enoxaparin sodium injection (0.8 ml, every 12 h) and warfarin (5 mg a day) were administered to prevent further vegetation generation. The diagnosis of *Staphylococcus epidermidis* endocarditis was confirmed by blood culture on day 4 after admission. Differential diagnoses included intracardiac thrombus, cardiac myxoma, non-bacterial thrombotic endocarditis and valve calcification.

**FIGURE 2:**
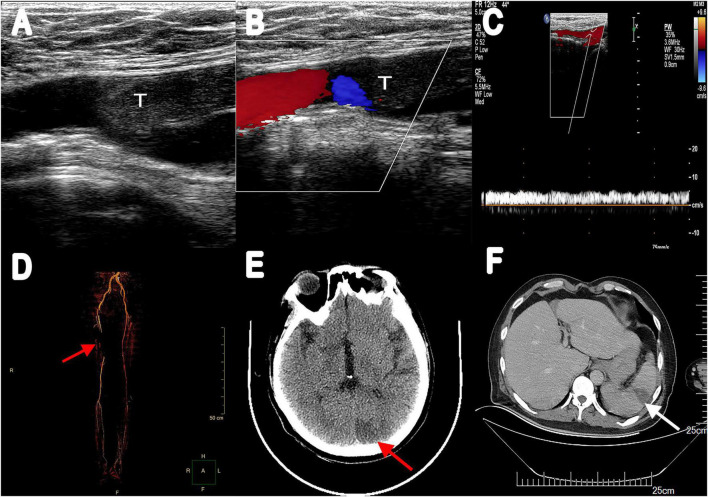
**(A)** Vascular ultrasonography showed hypoechoic filling echo was seen at the distal end of the right common femoral artery. **(B)** Color Doppler image displayed there was little color blood flow signal. **(C)** Abnormal spectrum morphology of posterior tibial artery with the significantly reduced blood flow velocity. **(D)** Multiple filling defects of the right femoral artery were observed in three-dimensional reconstruction (red arrow). **(E)** Enhanced CT scanning revealed a low-density lesion in the left occipital lobe (red arrow). **(F)** Abdominal enhancement CT showed wedge-shaped hypodense lesions under the capsule with no enhancement (white arrow). T, thrombus.

**FIGURE 3:**
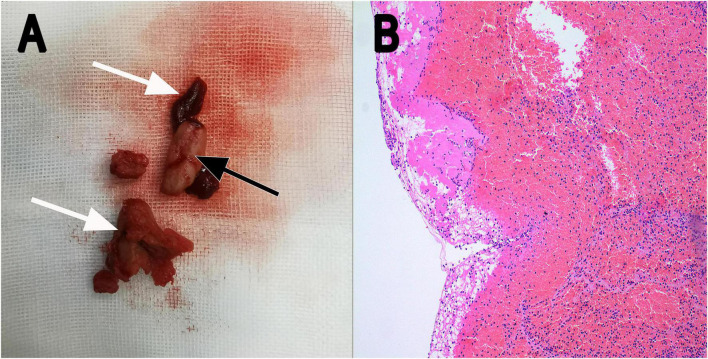
**(A)** Thrombotic substance and vegetations removed by surgery in gross appearance (white arrow: thrombotic substance; black arrow: vegetation). **(B)** Fiber-like substance and many red blood cells were seen under the microscope (H&E staining; magnification, ×10). H&E, hematoxylin and eosin.

The recommendation for surgery was considered inappropriate as the patient was still in the convalescent phase of Bentall surgery. Antibiotic treatment was continued. On day 21, he suffered from right lower extremity numbness without an apparent cause. The vascular ultrasound showed hypoechoic mass at the distal end of the right common femoral artery with a size of 38 mm × 11 mm without significant activity (Figure 2A), strongly suggesting right common femoral artery embolism, one of the complications of PVE. Color Doppler showed scattered color blood flow signal (Figure 2B) and delayed upstroke waveforms (Figure 2C). The embolism in the right femoral artery was also confirmed by enhanced CT reconstruction (Figure 2D). Our patient immediately underwent arteriotomy to remove the embolism. Macroscopic inspection revealed thrombotic substance and vegetations (Figure 3A). Fiber-like material and red blood cell aggregation were shown under the microscope (Figure 3B). Pathological examination confirmed the right lower extremity artery embolism.

On day 28, the patient presented with sudden convulsion. Enhanced CT scanning revealed left occipital cerebral embolism (Figure 2E). Deproteinized calf serum injection (0.8 g once a day) was given to improve brain blood circulation. He underwent a TTE reexamination on day 31, showing that aortic valve and tricuspid valve vegetations were still present while the aortic valve vegetation was smaller compared with the last time (Figure 1C). However, a new mitral valve regurgitation has emerged (Figure 1D). Unfortunately, he presented with decreased muscle tone in the right limb on day 35, and the subsequent CT scan showed another new spot of cerebral embolism.

Furthermore, the patient developed abdominal pain, and the CT scan revealed splenic infarction (Figure 2F) on day 40. After 7 weeks of antibiotic and anticoagulant therapy, The patient’s clinical symptom improved and body temperature was normal (36.5°C). Blood culture was negative. He was discharged after completing 45 days of hospitalization therapy and a follow-up 2 weeks later was advised. Oral warfarin (5 mg/d) was given once daily after discharge to maintain the international normalized ratio between 2 and 3 without significant bleeding tendency. Unfortunately, he died of cerebral infarction on day 55.

## Discussion

PVE is a rare but dangerous complication in patients after aortic valve replacement, with a high mortality rate of 20–40%. Moreover, 80% of death cases are associated with embolic events (1). There are few case reports on PVE complications after Bentall surgery. To our knowledge, this is the first report of the *Staphylococcus epidermidis* PVE complicated with multiple systemic embolisms early after a Bentall procedure.

Patients with cardiac structural abnormalities are vulnerable to IE, including congenital heart disease, prosthetic valve replacement, valvular stenosis, and regurgitation, leading to local hemodynamic abnormalities, where the endocardium is damaged under the constant shock of blood flow. In this scenario, the occurrence of bacteremia, caused by infected focus in other parts of the body, will promote IE. The most common organisms known to cause PVE are Staphylococcus (5). However, early infections (<1 year) caused by low pathogenic pathogens, such as *Staphylococcus epidermidis*, were rare. *Staphylococcus epidermidis* is an opportunistic pathogen mainly distributed in the intestine and has the possibility of invading the human body through soft tissue infection (3).

The most frequent symptom in patients with PVE is fever, arterial embolism, and heart failure. Systemic embolism is a serious complication of PVE, accounting for approximately 21.1% of patients with left-sided IE (6). Vegetation larger than 1 cm in diameter increases the incidence of embolization and mortality. Left-sided IE is always accompanied by splenic infarction and stroke (7, 8). However, concurrent embolization in the brain and spleen is rare (9). In our case, cerebral and splenic infarctions occurred successively within nine days, caused by the multiple shedding of the large vegetation.

Echocardiography is the preferred technique for the diagnosis of endocarditis. The most common echocardiographic finding is the presence of vegetation and/or abscess on prosthetic valves. New-onset valve regurgitation also indicates higher likelihood of endocarditis. Echocardiography is simple and easy to perform and enables the accurate assessment of the size, location, and morphology of vegetations. Echocardiography must be performed immediately as long as IE is suspected (10). In this case, the echocardiographic findings and blood culture results were quite unequivocal. Large, mobile vegetations are the prerequisite of arterial embolism in PVE.

The fibrinolysis and anticoagulation therapy for IE patients with vegetation remains controversial. The American College of Cardiology/American Heart Association guidelines recommend surgery or the use of slow-infusion, low-dose (25 mg of tissue-type plasminogen activator over 6–24 h without bolus) fibrinolysis protocol as initial treatment (11). It is worth noting that the risk of complications of fibrinolysis therapy is related to thrombosis area and previous stroke history. A thrombus area ≥1.6 cm^2^ has a complication rate of 47% (12). On the other hand, the European Society of Cardiology guidelines are more inclined to surgery (13). Our patient was not received thrombolysis due to the high risk of complications of thrombolytic therapy in this patient with large vegetation on his mechanical aortic valve (25 mm × 22 mm). In addition, some researchers suggest continuing anticoagulant therapy for patients with mechanical valve IE. However, the general recommendation is to discontinue all forms of anticoagulation for at least 2 weeks in patients with mechanical valve IE who have experienced a central nervous system embolic event. This time allows for thrombus organization and prevent the acute hemorrhagic transformation of embolic lesions. Special care should be taken when reintroducing anticoagulants. Intravenous unfractionated heparin is first applied until the activated partial thromboplastin time range from 50 to 70 s and then adjusted dose warfarin is used carefully (14). In our case, huge vegetation on the mechanical aortic valve and continued anticoagulant therapy after cerebral infarction together contribute to the risk of shedding of unstable vegetation, which is considered as the cause of failure of anticoagulation and subsequent recurrent embolism.

The PVE has attracted much more attention due to its high mortality rate. The earlier the diagnosis, the better the prognosis. A multidisciplinary team including cardiology, internal medicine, and infectious diseases should be jointly organized to manage patients with PVE. The absolute indication for early PVE surgery is *Staphylococcus aureus* infection to prevent brain embolization. Serving as the preferred treatment for PVE patients, surgery is also recommended for patients with hemodynamic instability, recurrent infection, or emboli. On the contrary, conservative treatment with antibiotics alone is often associated with adverse outcomes (15). Therefore, the prevention of PVE should be focused on the antibiotic prophylaxis before and after invasive procedures. Reducing perioperative invasive procedures (such as central venous access and catheterization) is another effective way to reduce bacteremia and subsequent valves contamination. Additionally, more frequent follow-up and monitoring are also necessary for the first year after replacement. Health education is equally important. Our patient was not a candidate for reoperation because of persistent fever and a history of surgery a month ago. Hence, he only received symptomatic treatment, which means the subsequent high risk of development of multi-organ embolisms or even death.

## Conclusion

We report a sporadic case of *Staphylococcus epidermidis* IE involving multiple valves and systemic embolism early after Bentall operation in a patient with Marfan’s syndrome. Continuous anticoagulation therapy after cerebral infarction may be the cause of deterioration. This case emphasizes that patients with early PVE may have poor prognosis and systemic embolization should be highly vigilant when PVE patients with large vegetations start to develop dyskinesia, abdominal pain, and limb numbness. Vascular ultrasound and echocardiography serve as the primary and reliable diagnostic methods in this situation.

## Data availability statement

The original contributions presented in this study are included in the article/supplementary material, further inquiries can be directed to the corresponding author.

## Ethics statement

This study was approved by the Ethical Committee of Shengjing Hospital of China Medical University. Written informed consent was obtained from patient’s next of kin.

## Author contributions

SW and XW drafted the manuscript and participated in cardiac ultrasonography. XY interpreted and revised the manuscript and conducted the vascular and cardiac ultrasound. WR and GS were involved in echocardiographic diagnosis. YH provided radiology images. HH was the surgeon in the case. All authors contributed to the study conception and design and approved the final manuscript.
